# Biochemical Interaction between Materials Used for Interim Prosthetic Restorations and Saliva

**DOI:** 10.3390/ma15010226

**Published:** 2021-12-29

**Authors:** Mihaela Pantea, Alexandra Ripszky Totan, Marina Imre, Alexandru Eugen Petre, Ana Maria Cristina Țâncu, Cristian Tudos, Alexandru Titus Farcașiu, Mihai Butucescu, Tudor Claudiu Spînu

**Affiliations:** 1 Department of Fixed Prosthodontics and Occlusology, Faculty of Dental Medicine, “Carol Davila” University of Medicine and Pharmacy, 17-23 Plevnei Street, 020221 Bucharest, Romania; mihaela.pantea@umfcd.ro (M.P.); alexandru.petre@umfcd.ro (A.E.P.); tudor.spinu@umfcd.ro (T.C.S.); 2 Department of Biochemistry, Faculty of Dental Medicine, “Carol Davila” University of Medicine and Pharmacy, 17-23 Plevnei Street, 020021 Bucharest, Romania; alexandra.totan@umfcd.ro; 3 Department of Complete Denture, Faculty of Dental Medicine, “Carol Davila” University of Medicine and Pharmacy, 17-23 Plevnei Street, 020221 Bucharest, Romania; marina.imre@umfcd.ro; 4 Resident in General Dentistry, Emergency Hospital of Saint Pantelimon, 021661 Bucharest, Romania; cristian.tudos@rez.umfcd.ro; 5 Department of Removable Prosthodontics, Faculty of Dental Medicine, “Carol Davila” University of Medicine and Pharmacy, 17-23 Plevnei Street, 020221 Bucharest, Romania; alexandru.farcasiu@umfcd.ro; 6 Department of Operative Dentistry, Faculty of Dental Medicine, “Carol Davila” University of Medicine and Pharmacy, 17-23 Plevnei Street, 020221 Bucharest, Romania

**Keywords:** dental materials, interim prosthetic restorations, saliva, oxidative stress, biocompatible materials

## Abstract

The purpose of this study was to analyze the oxidative stress level and inflammatory status of saliva in the presence of certain materials used for obtaining interim prosthetic restorations. Four types of interim resin materials were investigated: a pressure/heat-cured acrylic resin (Superpont C+B, SpofaDental a.s Czech Republic, /KaVo Kerr Group), a milled resin (Telio CAD polymethyl methacrylate, Ivoclar Vivadent AG, Liechtenstein), a 3D printed resin (NextDent C&B MFH, NextDent by 3D Systems, the Netherlands), and a pressure/heat-cured micro-filled indirect composite resin (SR Chromasit, Ivoclar Vivadent AG, Liechtenstein). The disk-shaped resin samples (30 mm diameter, 2 mm high) were obtained in line with the producers’ recommendations. The resulting resin specimens were incubated with saliva samples collected from twenty healthy volunteers. In order to analyze the antioxidant activity of the tested materials, certain salivary parameters were evaluated before and after incubation: uric acid, gamma glutamyl transferase (GGT), oxidative stress responsive kinase-1 (OXSR-1), and total antioxidant capacity (TAC); the salivary levels of tumor necrosis factor (TNFα) and interleukin-6 (IL-6) (inflammatory markers) were measured as well. The obtained results are overall favorable, showing that the tested materials did not cause significant changes in the salivary oxidative stress level and did not influence the inflammatory salivary status.

## 1. Introduction

Interim prosthetic restorations are essential for obtaining a predictable and personalized final prosthetic outcome; this type of restoration has alternatively been referred to as “provisional”, “temporary”, or “transitional” restoration. By definition, these restorations should have a limited lifespan in prosthetic therapy. However, interim restorations are designated for multiple, diverse clinical cases: emergencies, temporization (post-surgical prostheses; prostheses applied between tooth extraction and implant placement; prostheses applied after implant placement; fixed space-maintainers; and prosthetic systems for periodontal splinting), and the testing of certain parameters (new vertical occlusal dimension; new static and dynamic occlusal scheme; aesthetic changes in the frontal area of dental arches; and chewing pattern). Therefore, interim dental restorations can last in the oral cavity from a few weeks to a few months (or even one year). As a consequence, apart from the protection of soft and hard oral tissues during the manufacture of the definitive prostheses, the interim prostheses favor both the establishing of a correct diagnostic as well as the achievement of good aesthetics and oral functionality with the definitive prostheses. Additionally, interim prostheses are used as an efficient communication tool at different levels, while contributing to patient satisfaction, comfort, and confidence. However, the protection and healing of dental, periodontal, and mucosal tissues represent one of the main objectives of interim prosthetic restorations. In fact, interim prosthetic restorations represent veritable “instruments”, playing an important role in the dental-maxillary apparatus adaptation and reshaping, especially in the complex oral rehabilitation cases associated with their extended use. This is due to the fact that interim prosthetic restorations allow for the testing of different functional parameters, facilitate and guide the healing process of peri-implant gingival tissue, and contribute to the healing and reshaping of periodontal tissues. Therefore, it is imperative that the materials from which the interim restorations are obtained should prove, first of all, to have very good biocompatibility [1,2,3].

Certain aspects concerning the conventional and modern materials used for manufacturing interim prosthetic restorations represent important topics in present-day scientific research: the dimensional accuracy and mechanical behavior (compression strength, flexural strength, tensile strength, and wear resistance) [4,5,6,7,8,9]; color stability and reparability [10,11,12]; and chemical composition and biocompatibility issues (such as cytotoxicity, the materials’ interactions with oral epithelial cells, fibroblasts or dental pulp cells monomer release bacterial adhesion, biofilm formation, antimicrobial activity, and the materials’ interactions with saliva) [13,14,15,16,17,18,19,20,21,22,23,24]. With regard to biocompatibility, the results of various tests performed on saliva samples can contribute to establishing a diagnosis in diverse local or systemic diseases and to monitoring physiological or pathological conditions. Saliva samples, considered a valuable source for biomarkers acquisition, can be easily and non-invasively collected; an analysis of these biomarkers can reveal important details on the metabolic, immunological, hormonal, and nutritional status, or even on individual stress level, of patients [25,26,27,28,29]. Certain salivary biomarkers can be used for analyzing the salivary oxidative stress response (redox biomarkers) or the inflammatory salivary status (i.e., cytokines). Thus, salivary uric acid can represent a valuable biomarker when studying the oxidative stress. Gamma glutamyl transferase (GGT) plays a key role in regulating the intracellular glutathione levels and maintains the cellular redox homeostasis [30], while oxidative stress responsive kinase-1 (OXSR1), apart from its multiple functions in essential cellular processes, reacts to oxidative stress. Alternatively, cytokines significantly contribute to inflammatory responses, and their expression is influenced by the presence of foreign bodies, pathogenic bacteria, or chemicals released by different materials, including interim resin materials [31]. Cytokines can alter the matrix metalloproteinase expression, which plays major roles in the collagen remodeling of the gingival extracellular matrix (ECM) [32,33]. At the same time, certain cytokines that are related to inflammation can be identified in saliva (interleukin-1/IL-1-beta (β), interleukin-6/IL-6, interleukin-8/IL-8, and tumor necrosis factor/TNF-α) [34,35]. IL-6 is a cytokine capable of mediating inflammation by strengthening the local defense and stimulating an immune response, while TNFα is a cytokine used by the immune system for cell signaling.

To our knowledge, few scientific studies were dedicated to topics related to the influence exercised by the materials used for obtaining interim prosthetic restorations on the salivary oxidative stress response and inflammatory salivary status.

Given this context, the present study investigates aspects related to the biochemical interactions between saliva and different interim prosthetic materials: a pressure/heat-cured acrylic resin (Superpont C+B, SpofaDental a.s., Jicin, Czech Republic, KaVo Kerr Group), a milled resin (Telio CAD polymethyl methacrylate, Ivoclar Vivadent AG, Schaan, Liechtenstein), a 3D printed resin (NextDent C&B MFH, NextDent by 3D Systems, Soesterberg the Netherlands), and a pressure/heat-cured micro-filled indirect composite resin (SR Chromasit, Ivoclar Vivadent AG, Liechtenstein); the influence of the above-mentioned materials on the salivary levels of various oxidative stress parameters (uric acid, gamma glutamyl transferase (GGT), oxidative stress responsive kinase-1 (OXSR-1), and total antioxidant capacity (TAC)) and on the levels of certain salivary markers for inflammation (tumor necrosis factor (TNFα) and interleukin-6 (IL-6)) were assessed, and the obtained results were statistically analyzed.

## 2. Materials and Methods

This study was approved by the Scientific Research Ethics Committee of “Carol Davila” University of Medicine and Pharmacy, Bucharest, Romania (protocol code: PO-35-F-03; number: 30929), and was conducted according to the guidelines of the Declaration of Helsinki, the Belmont Report, the Council for International Organizations of Medical Sciences (CIOMS), and the International Conference on Harmonisation Good Clinical Practice (ICH-GCP) Guideline. Written informed consent was duly signed by all subjects involved in the study.

### 2.1. Fabrication of Resin Specimens

The following dental materials used for manufacturing the interim prosthetic restorations were selected in order to be tested during this study: a pressure/heat-cured acrylic resin (Superpont C+B, SpofaDental a.s., Jicin, Czech Republic, KaVo Kerr Group), a milled resin (Telio CAD polymethyl methacrylate, Ivoclar Vivadent AG, Schaan, Liechtenstein), a 3D printed resin (NextDent C&B MFH, NextDent by 3D Systems, Soesterberg, The Netherlands) and a pressure/heat-cured micro-filled indirect composite resin (SR Chromasit, Ivoclar Vivadent AG, Schaan, Liechtenstein). This study included both conventional (composite and acrylic resins) and modern (3D printed and milled resins) interim prosthetic materials. The main criteria for the selection of the dental materials used in this study included the following aspects: the materials should be recommended for obtaining interim dental prostheses; the materials should allow the fabrication of both short- and long-term interim dental prostheses; the materials should be commercially available; and the materials should be acknowledged for their frequent use in daily practice in the domain of prosthetic dentistry.

The disk-shaped samples were designed to be circular in the cross section and 2 mm thick with a 30 mm diameter.

**Figure 1:**
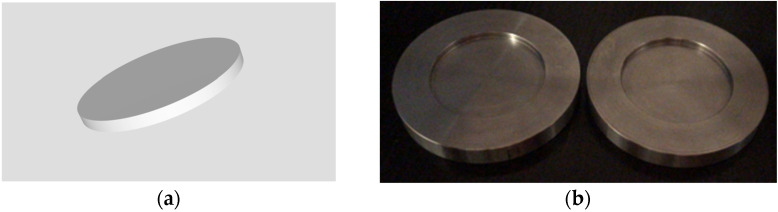
(**a**) A 2D frame corresponding to the digital design of the milled PMMA and 3D printed specimens; (**b**) conformers used in the manufacturing of the indirect composite resin and pressure/heat-cured acrylic resin specimens.

The polymethyl methacrylate (PMMA) milled specimens and the 3D printed ones were manufactured by using CAD/CAM (computer aided design/computer aided manufacturing) technology. The disk were designed using the Fusion 360 CAD software (Autodesk, Mill Valley, CA, USA); an STL file (Figure 1a) containing the specific design for the samples (with the established dimensions) was generated and exported to a 3D printer (NextDent 5100, NextDent by 3D Systems, Soesterberg, The Netherlands) in order to fabricate the printed resin specimens, and to a milling machine (PrograMill PM 7, Ivoclar Vivadent AG, Schaan, Liechtenstein), for obtaining the milled PMMA ones.

The indirect composite resin and the pressure/heat-cured acrylic resin specimens were fabricated by using metal alloy (Cr–Co) conformers; these conformers were produced by employing CAD/CAM technology: they were digitally designed and then obtained by subtractive manufacturing (milling) (Figure 1b). The shape and dimensions of the conformers were established with the aim that the internal diameter should be 30 mm while the height of the internal edges should be 2 mm; this allowed the fabrication of the composite resin and acrylic resin specimens with the above-mentioned dimensions.

In order to fabricate the indirect composite resin specimens, with the aim of ensuring a smooth detachment, a separating solution Isodent (SpofaDental a.s., Jicin, Czech Republic, KaVo Kerr Group) was applied inside the conformers using the appropriate dental instruments (a sterile dental composite non-stick spatula and pluggers). Upon insertion and levelling of the composite resin, a small board of sterile glass was slightly pressed against the conformer in order to eliminate the material in excess and obtain the flattest possible external surface. The composite resin was polymerized in the Ivomat IP3 (Ivoclar Vivadent AG, Schaan, Liechtenstein), oven at 120 °C under 6 bar pressure for 7 min. The resin composite specimens were finished and then polished manually with a low-speed handpiece, using silicone polishing cups, brushes, and Ivoclar universal polishing paste (Ivoclar Vivadent AG, Schaan, Liechtenstein). With regard to the pressure/heat-cured acrylic resin specimens, they were obtained by mixing the powder with an appropriate quantity of liquid/monomer (in a ratio of 3:1/3 parts of powder to 1 part of liquid) and inserting the resulting paste into the conformer previously isolated with Isodent (SpofaDental a.s., Jicin, Czech Republic, KaVo Kerr Group). The specimens were maintained at 93 °C under 0.6 MPa pressure for 25 min in order to achieve final polymerization, then finished, and polished manually.

**Figure 2:**
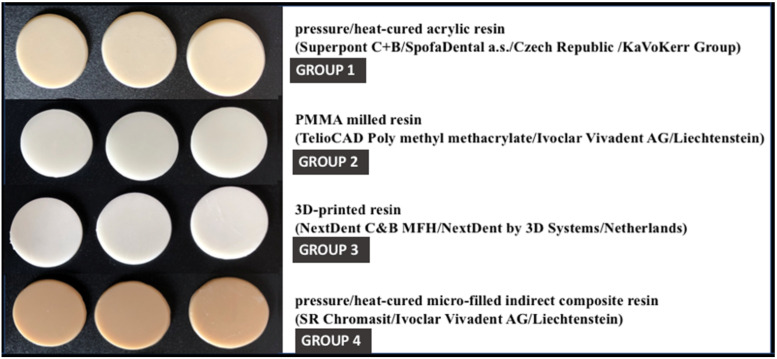
The obtained resin specimens.

Three specimens of each previously presented material were fabricated, resulting in a total number of 12 specimens (see Figure 2). Four groups of investigated materials were created as follows:

- Group 1—corresponding to pressure/heat-cured acrylic resin;
- Group 2—corresponding to milled PMMA resin;
- Group 3—corresponding to 3D printed resin;
- Group 4—corresponding to indirect composite resin.

### 2.2. Collection of Saliva Samples

Regarding the participants in this study, the inclusion criteria were as follows: females or males aged from >18 to 65 years; psychological competence; understanding capacity; legal competence; favorable general health status (absence of systemic diseases); complete dental arches—presenting, at most, resin composite fillings or ceramic crowns on natural teeth; absence of active periodontal disease; absence of oral mucosal lesions; as well as non-smokers. The exclusion criteria were: lack of cooperation with the medical staff; any communication, neurological, and/or cognitive impairment; unwilling to undergo oral examinations and proposed protocols or to sign the consent form; partial or total edentulous state; presence of active periodontal disease; pregnancy; smoking, drug-use or alcohol abuse; presence of fixed or mobile prostheses supported on natural teeth or on implants; presence of oral mucosal lesions; presence of orthodontic appliances; medications with xerogenic effects; as well as salivary gland dysfunctions or salivary glands extirpation.

The saliva samples were collected from 20 healthy volunteers (*n* = 20) selected from the patients of the Prosthodontics Clinic, Faculty of Dentistry, “Carol Davila” University of Medicine, Bucharest, Romania. Given the present SARS-CoV-2 pandemic situation, in order to prevent and limit the extension of the COVID-19 infection, a pre-established protocol was followed. Saliva samples were collected according to the strict preventive measures adopted due to the SARS-CoV-2 pandemic. Participants in this scientific research voluntarily accepted to be included in the study and signed an informed consent. All participants were instructed not to eat, brush their teeth, or use mouth rinse for at least 2 h prior to sample collection and were asked to rinse their mouth with distilled water immediately prior to sample collection. Samples of 1.0–2.0 mL of unstimulated saliva were obtained from each of the 20 volunteers between 9 am and 10 am, so that possible circadian variation would be reduced. All saliva samples were collected in special sterile test containers. Within a maximum of 1h from saliva collection, all saliva sample containers were transported to the biochemistry laboratory in a special insulated cooler bag at a low temperature (2–40 °C). The resin specimens for fabricating interim prosthetic restorations were also transported to the biochemistry laboratory in appropriate conditions.

### 2.3. Preparation of Saliva Samples and Biochemical Testing

Control saliva samples were immediately centrifuged for 10 min at 3000 rpm to remove bacterial and cellular debris. Saliva samples designated for testing were immediately incubated for 12 h, at 37 °C, with samples of resin materials (one dental material sample/500 μL of saliva). After the incubation period, the saliva samples were centrifuged for 10 min at 3000 rpm, in order to remove bacterial and cellular debris. All of the biochemical determinations corresponding to this study were performed using the supernatant. Salivary uric acid, GGT, albumin, OXSR-1, TNFα, and IL-6 were assayed both in the incubated and control samples immediately after centrifugation. The concentrations of all salivary parameters were expressed relative to the salivary concentration of albumin in order to avoid the salivary flow influence. Salivary albumin, uric acid and GGT were measured using analyzing kits (Biosystems, Barcelona, Spain), on a biochemistry automatic analyzer A25 (Biosystems, Barcelona, Spain), according to the suppliers’ instructions. For the salivary OXSR-1 measurements, we used ELISA analyzing kits (Blue Gene, Shanghai, China). IL-6 was measured using an automatic chemiluminescence analyzer (Maccura 1200 PLUS, Chengdu, China).

### 2.4. Statistical Analysis

All data collected in the study was analyzed using IBM SPSS Statistics 25 and illustrated using Microsoft Office Excel/Word 2013. Quantitative variables were tested for normal distribution using the Shapiro–Wilk Test and were written as averages with standard deviations. Quantitative variables with non-parametric distribution were tested between groups using Kruskal–Wallis H tests. Quantitative variables with parametric distribution were tested between groups using One-Way ANOVA and Welch’s ANOVA tests according to Levene’s test results. Post-hoc tests, such as the Tukey HSD, Games–Howell, and Dunn–Bonferroni, were performed to further analyze the results obtained in the quantitative variables’ testing.

## 3. Results

The analysis of the interaction between the materials designated for fabricating interim prosthetic restorations and the salivary environment consisted of evaluating their antioxidant activity by determining the value of certain parameters: salivary redox biomarkers (uric acid, GGT, oxidative stress responsive kinase-1 (OXSR-1), and total antioxidant capacity (TAC)) and the influence of these materials on the inflammatory salivary status, assessed by determining the levels of tumor necrosis factor (TNFα) and interleukin 6 (IL-6). The statistical analysis revealed the following aspects: 

The data in Table 1 and Table 2 present a description and comparison of the biochemical parameters for the analyzed materials. Significant differences were observed between materials for the before incubation ratios of IL-6/albumin (*p* = 0.003) and TNFα/albumin (*p* = 0.003) and the after incubation ratios of uric acid/albumin (*p* = 0.032), IL-6/albumin (*p* = 0.004), and TNFα/albumin (*p* = 0.001).

**Figure 3:**
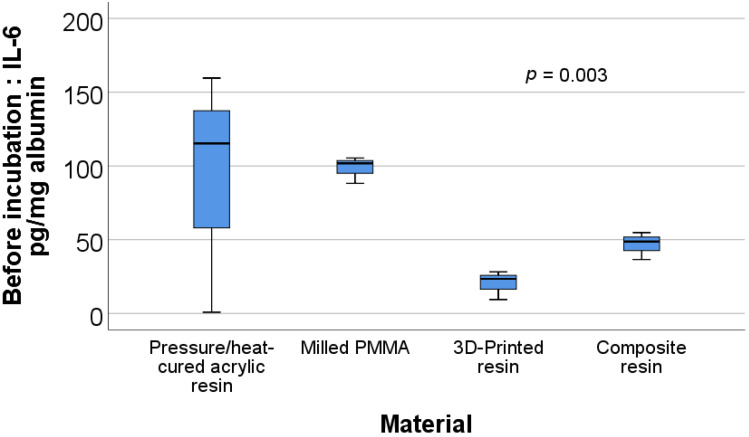
Box plot representation of the comparison between IL-6/albumin ratio values—before incubation for each material type.

Figure 3 shows the box plot representation of the IL-6/albumin ratio values for each of the material types before incubation. Post-hoc tests show that the values for the milled PMMA material (98.47 ± 9.05) were significantly higher than those for the 3D printed resin (20.33 ± 9.76) (*p* = 0.002) or composite resin (46.69 ± 9.302) (*p* = 0.008).

**Figure 4:**
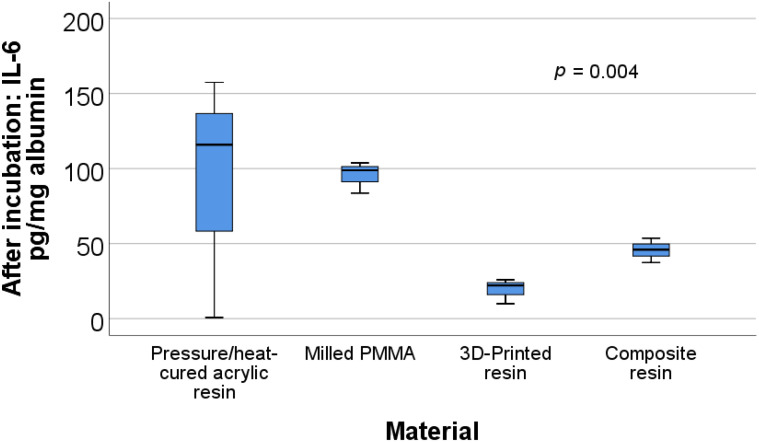
Box plot representation of the comparison between IL-6/albumin ratio values—after incubation for each material type.

Figure 4 shows the box plot representation of the IL-6/albumin ratio values for each material type after incubation. Post-hoc tests show that the values for the milled PMMA material (95.41 ± 10.53) were significantly higher than those for the 3D printed resin (19.3 ± 8.35) (*p* = 0.003) or composite resin (45.64 ± 8.07) (*p* = 0.012).

**Figure 5:**
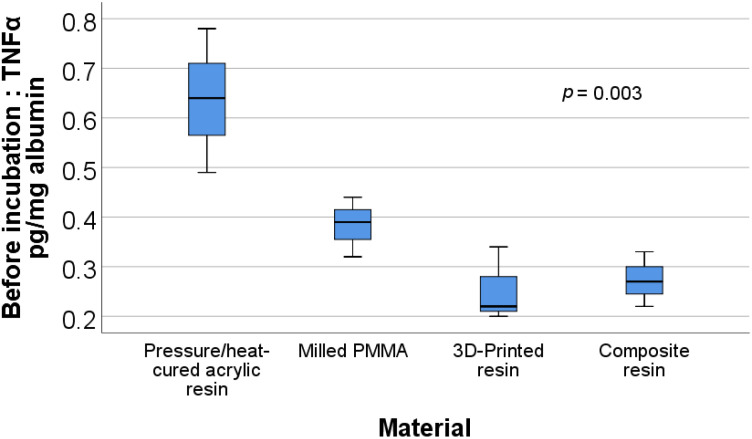
Box plot representation of the comparison between TNFα/albumin ratio values—before incubation for each material type.

Figure 5 shows the box plot representation of the TNFα/albumin ratio values for each material type before incubation. Post-hoc tests show that the values for the pressure/heat-cured acrylic resin (0.636 ± 0.145) were significantly higher than those for the milled PMMA (0.383 ± 0.06) (*p* = 0.038), 3D printed resin (0.253 ± 0.075) (*p* = 0.004), or composite resin (0.273 ± 0.055) (*p* = 0.005).

**Figure 6:**
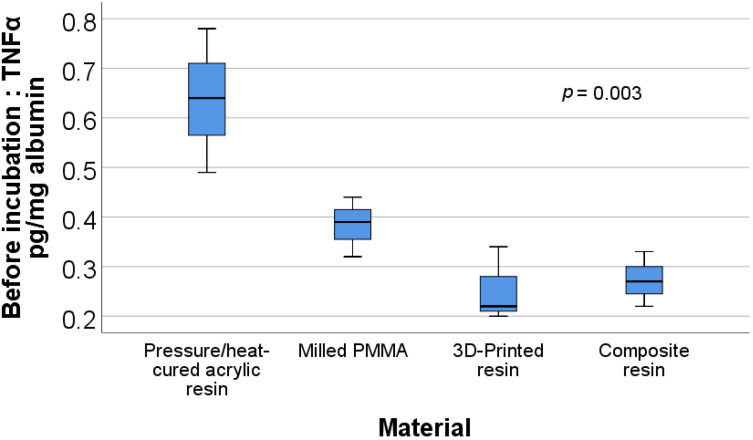
Box plot representation of the comparison between TNFα/albumin ratio values—after incubation for each material type.

Figure 6 shows the box plot representation of the TNFα/albumin ratio values for each material type after incubation. Post-hoc tests show that the values for the pressure/heat-cured acrylic resin (0.616 ± 0.09) were significantly higher than those for the milled PMMA (0.37 ± 0.06) (*p* = 0.008), 3D printed resin (0.25 ± 0.05) (*p* = 0.001), or composite resin (0.286 ± 0.055) (*p* = 0.001).

**Figure 7:**
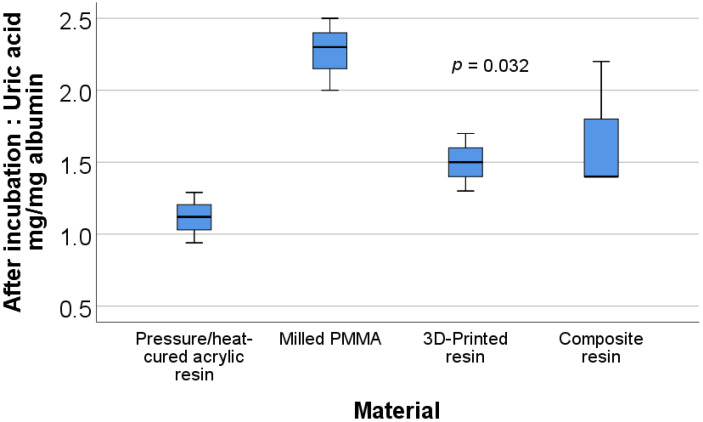
Box plot representation of the comparison between uric acid/albumin ratio values—after incubation for each material type.

Figure 7 shows the box plot representation of the uric acid/albumin ratio values for each material type after incubation. Post-hoc tests show that the values for the pressure/heat-cured acrylic resin (mean rank = 2.00) were significantly lower than those for the milled PMMA (mean rank = 10.67) (*p* = 0.019).

## 4. Discussion

The present study focused on analyzing the biochemical interactions between saliva and certain dental materials used for manufacturing interim prosthetic restorations. With the aim of augmenting the clinical relevance of the results of this study, the selection of materials for the in vitro testing included two conventional material types used for fabricating interim prosthetic restorations: a pressure/heat-cured micro-filled indirect composite resin (SR Chromasit, Ivoclar Vivadent AG, Schaan, Liechtenstein) and a pressure/heat-cured acrylic resin (Superpont C+B, SpofaDental a.s., Jicin, Czech Republic, KaVo Kerr Group), as well as two modern material types: one obtained by additive manufacturing (NextDent C&B MFH, NextDent by 3D Systems, Soesterberg, The Netherlands) and one by subtractive technology (Telio CAD polymethyl methacrylate, Ivoclar Vivadent AG, Schaan, Liechtenstein). The investigated materials have already demonstrated their clinical applicability and are widely used in the field of prosthetic dentistry. However, few scientific studies that focus on the impact of interim prosthetic materials on salivary oxidative stress and on inflammatory salivary status are found in the scientific literature. The obtained biochemical data of the present study show that the saliva incubation in the presence of the tested materials does not generate significant modifications in the levels of the salivary oxidative stress and inflammatory salivary status, thus contributing to highlight the biocompatibility of the tested interim prosthetic materials.

Dental literature includes relevant studies regarding the biocompatibility of the materials used for manufacturing interim prosthetic restorations, with a general focus on the interaction between these materials and oral cells (fibroblasts, epithelial cells, or dental pulp cells); the monomer release; or biofilm formation [36,37,38,39,40,41,42,43,44]. Few studies are dedicated to the influence exercised by interim prosthetic materials on the salivary redox status or inflammatory salivary status. However, our results are in line with the outcome of other scientific research, which confirms the biocompatibility of such materials and approves, to a large extent, their clinical use. In fact, the majority of the dental studies we have consulted claim that the materials used for fabricating interim prosthetic restorations cannot alter the physiological status of the oral environment thanks to their good biocompatibility. Moreover, modern materials (3D printed and milled PMMA resins) present a higher biocompatibility compared to the conventional interim materials. For instance, Gonçalves et al. (2016) [36] showed that the tested bis-acryl resins (Protemp 4, 3M ESPE, Brazil and Luxatemp Star, DMG, Germany) were cytocompatible with human gingival fibroblasts, thus suggesting that both materials are suitable for use in close contact with human gingival tissues. Shim et al. (2019) [37], analyzing the responses of human gingival fibroblast (HGF-1) in contact with diverse prosthetic interim materials, suggested that CAD/CAM technology and indirect fabrication (in the dental laboratory) of interim prosthetic restorations are recommended in order to prevent residual monomer elution and achieve high cell attachment. Souza et al. (2020) [38] also demonstrated that the CAD/CAM acrylic resin was the most compatible with the oral epithelial cells in comparison with the conventional acrylic and bis-acrylic resins. Park et al. (2020) [39] recommended the resins obtained by additive manufacturing for fabricating interim prosthetic restorations over using the auto-polymerized acrylic resins, based on the comparative evaluation of their cytotoxicity (cellular attachment and cell proliferation of mice gingival fibroblasts). Campaner et al. (2020) [40] concluded that the CAD/CAM obtained resins could be considered the most suitable materials for fabricating interim restorations; in this study, the tested auto-polymerized acrylic resin (Alike, Reliance Dental Mfg Co., Worth, IL, USA) and bis-acrylic resin (VIPI Cor, VIPI Industries, Toledo, Spain) induced the greatest adverse effects on mice gingival fibroblasts while the CAD/CAM nano ceramic resin (LAVA Ultimate 3 M ESPE Dental Products, St. Paul’s, MN, USA) and the prefabricated polymer block (Telio CAD, Ivoclar Vivadent AG, Schaan, Liechtenstein) were the most cytocompatible materials and induced the lowest production of IL-6, IL-1β, and TNF-α.

The milled PMMA, which is an interim prosthetic material, was compared even with ceramic-based materials that are dedicated for final prosthetic restorations, as in complex prosthetic rehabilitations or when some unpredictable situations may occur (quarantine, delays, illness, etc.). Interim prosthetic restorations must be designed in such a way that they withstand in the oral cavity for a long time without affecting the surrounding tissues. The biocompatibility of the milled PMMA Vita CAD-Temp (VITA Zahnfabrik, Bad Säckingen, Germany) and of several CAD/CAM ceramic materials (IPS e.max^®^ CAD, Ivoclar Vivadent AG, Schaan, Liechtenstein), VITA YZ T (Vita Zahnfabrik, Bad Säckingen, Germany), Celtra Duo (Degudent GmbH, Hanau-Wolfgang, Germany)) with human gingival fibroblasts were evaluated in a study by Rizo-Gorrita et al. (2019) [41]. This study demonstrated good biocompatibility levels in all of the analyzed materials, even if the lithium disilicate ceramics revealed better cell responses than the polymers—in terms of cell viability and collagen type I secretion. The results of another in vitro study [42] by Herráez-Galindo et al. (2017), which evaluated the fibroblastic behavior on milled PMMA (Vita CAD-Temp, VITA Zahnfabrik, Bad Säckingen, Germania) and on lithium disilicate (IPS e.max® CAD, Ivoclar Vivadent AG, Schaan, Liechtenstein), similarly pointed out that the milled PMMA demonstrated a cellular behavior that is comparable to that of the lithium disilicate. No statistically significant differences were found for the majority of the studied parameters (cell proliferation, average nuclei size, and area covered by cell nuclei), with the exception of the cytoskeleton length of the fibroblasts; the latter was found higher for the PMMA, and, therefore, it was concluded that fibroblasts expand better over this material. Taking into account these results, the authors suggested that the milled PMMA, as a material for interim prosthetic restorations, could contribute to gingival healing and reshaping, which is crucial/essential mainly in interim implant supported restorations.

The scientific research on interim prosthetic materials also addresses their compatibility with human dental pulp cells. Jun et al. (2017) [45] studied in vitro the cytotoxicity and the pro-inflammatory cytokine expression of interim resin materials on human dental pulp stem cells. The authors reported that possible pulp damage caused by released toxic components should be considered, especially when “chemical-activated provisional resin materials are applied to extensively prepared teeth”. Lee et al. (2017) [46], present similar results, confirming that extensive teeth preparations, that imply a significant reduction in hard dental tissue exposes the pulp tissue to possible damage during the direct interim prosthetic phase, mostly when chemical-activated resins are used.

Another aspect to be considered, but at the same time is debatable with regard to the biocompatibility of interim prosthetic materials, is the release of residual monomers. Back in 2013, Ivković et al. [47] presented the conclusions of their study on the acrylic monomer used in acrylic dental resins and its adverse reactions. Acrylic-based resins, intensively used in dental practice as the basic materials for interim prosthetic restorations; orthodontic appliances; occlusal splints; and removable, partial, or complete dentures raise the issue of compatibility with the oral environment, especially with regard to their manufacturing process and polymerization, but also in terms of their biodegradability in the oral environment. The authors noted that the manufacturing process and polymerization of conventional acrylic-based resins (auto-polymerized and pressure/heat-cured acrylic resins) can influence their cytotoxic effect by way of various aspects, such as: the method applied for obtaining the resins or the duration of the storage of the materials (powder and liquid) used for fabricating the final product—the resin, the powder/liquid ratio used for mixing, polymerization conditions, specific polymerization type, and duration. Alternatively, several factors lead to biodegradation: saliva characteristics (pH, fluid/viscous saliva, and rich/reduced salivary flow); chewing pattern (lateral or vertical chewing pattern, uni- or bilateral chewing, bite force symmetry, chewing time, and rhythm); stability of oral microbiota; or diet type. The consequences of both the manufacturing and polymerization processes, as well as the resin’s biodegradation, include various adverse effects on the oral health (irritation, inflammation, and allergic responses of the oral cavity tissues) due mainly to the release of potential cytotoxic components from the polymer network. Observations similar to the above-presented findings are also contained in the relatively recent study of Bandarra et al. (2020) [48], which evaluated the cytocompatibility and the neurotoxic potential of the monomers for three interim conventional restoration materials, (Tab 2000, Kerr, USA (methyl methacrylate based), ProTemp 4, 3M, USA (bis-acrylic based), and Structur 3, Voco GmbH, Germany (urethane dimethacrylate/UDMA based)). The results of this study suggest that urethane dimethacrylate–based resin, even at low concentrations, may cause adverse local (oral) side effects and may have neurotoxic potential. Nevertheless, numerous studies in recent dental literature state that modern polymer interim prosthetic materials, obtained by additive technology or subtractive technology, demonstrate higher stability and resistance and lower elution of residual monomer than conventional polymers. For instance, the study performed by Engler et al. (2020) [49] aimed to analyze the residual monomer elution of nine polymers (obtained by conventional and CAD/CAM techniques) during artificial aging (the polymer samples were kept in distilled water for 60 days at 37 °C). The maximum registered residual elution was estimated to be under the accepted standards (ISO 20795-1 standard), which is—obviously—encouraging.

The rationale for the selection of the interim prosthetic materials used in this study includes the fact that the modern ones, obtained by additive and subtractive technologies, exhibit great potential for development in the field of dentistry, and, compared to the conventional ones, show distinct advantages (low material waste, easy mass customization, accelerated manufacturing process, accuracy, and reduced risk of cross-contamination via digital work-flow). Conversely, the tested conventional interim prosthetic materials have already proven their valuable properties (easy manipulation and repairing, good aesthetics, low cost, and clinically accepted biocompatibility). The comparative analysis of the investigated materials highlights the complex way in which they interact with the oral environment. Moreover, it is acknowledged that the interim prostheses are not restricted to acrylic and composite resins but could also be designed with a metal infrastructure, which is especially recommended in complex clinical cases when these restorations have to remain in situ for a longer period [50,51,52]. The infrastructure is veneered with acrylic or composite resins, resulting in a fixed resin–metal interim prosthesis. As an example, in this study, the tested indirect composite resin is used for obtaining metal-supported interim prostheses or long-term interim prostheses, when combined with a fibre-reinforced composite framework material (Vectris, Ivoclar Vivadent, Liechtenstein).

Our study shows that during saliva incubation in the presence of the tested materials (pressure/heat-cured acrylic resin, milled PMMA, 3D printed resin and pressure/heat-cured micro-filled indirect composite resin), no significant modifications of the levels of salivary oxidative stress were noted. However, the results indicate certain differences in the distribution of values of the analyzed parameters, depending on the material type. A statistical analysis reveals the fact that the distribution of the values corresponding to IL-6, before and after incubation, differ depending on the material type, e.g., the values for the milled PMMA were significantly higher than those for the 3D printed or composite resins. Additionally, the distribution of the values corresponding to the TNFα acid, before and after incubation, also differ depending on the material type, as the values corresponding to the pressure/heat-cured acrylic resin were significantly higher than those corresponding to the milled PMMA, 3D printed or composite resins. The above-mentioned findings are not clinically relevant as they indicate only discreet differences in the effects of these materials on the salivary environment. Nevertheless, these last remarks represent a signal similar to the ones launched by several other studies, which indicate that the interim prosthetic materials can present slight disadvantages with regard to the response that they generate in the body [47,48,53,54]. In this respect, back in 2009, Ulker et al. [53] advised that some interim prosthetic materials might have cytotoxic effects on fibroblasts and should be carefully selected for clinical applications. More recently, the cytotoxic effects of new generation all-ceramic (such as Lava Ultimate, VITA Mark II, InCoris TZI, IPS e.max^®^ CAD, VITA Suprinity, Cerasmart, and IPS Empress CAD) and interim materials (such as Protemp 4, Telio CAD, CAD-Temp, Telio Lab, Temdent Classic, and Telio CS C&B) on mice fibroblast cells were also studied by Atay et al. (2109) [54]. The results of this study revealed that, although the materials “display slight cytotoxicity values, the results are still within the reliable range, and they can safely be used in clinical conditions”. The authors of a recent study by Giti et al. (2021) [55] also state that the conventional and 3D printed resins were not cytotoxic to human gingival fibroblast-like cells, yet, the resins obtained by using subtractive method “were slightly cytotoxic”. Their results additionally showed that surface roughness was the highest for the conventional resin while the 3D printed resin presented the most plaque accumulation and lowest cytotoxicity.

Apart from the above-presented elements, it has been acknowledged that, in order for a material to prove of a good biocompatibility, it must, first of all, have appropriate chemical composition. The chemical composition of conventional interim materials is based on monomethacrylates (or acrylic resins) and on dimethacrylates or bis-acryl/composite resins (such as bisphenol A-glycidyl dimethacrylate and urethane dimethacrylate); alternatively, the chemical composition of 3D printed resins has not been fully disclosed by manufacturers [1,56,57]. At the same time, the dimensional accuracy of 3D printed prosthetic restorations depends on diverse factors, such as the position and angle of the restoration on the printing platform, the amount of supportive material, the laser speed, the material’s shrinkage rate, the post-processing procedures, and the type of design software. In addition to the above, the selection of interim restorations—with regard to their manufacturing process and materials—should be individualized considering the following factors: chewing forces, chewing pattern, parafunctions, length of edentulous spans, the age of edentulism, and the type of prosthetic restoration (supported by natural teeth or by dental implants) [58,59,60].

Among the limitations in the present study, we include a reduced number of investigated materials, a reduced number of material and saliva samples, and a short incubation time; further studies should include the assessment of more salivary parameters as well. It should be noted that the design of this study was influenced by the present SARS-CoV-2 pandemic situation. Taking into account the multitude of elements defining the biocompatibility of conventional and modern interim prosthetic resins, we can conclude that a comparative evaluation of the interactions between these resins and the salivary environment, along with their proper clinical selection, represent complex, particularly challenging processes [1,3,61,62,63,64]. As a consequence, further in vitro and in vivo studies are needed in order to enhance the interim prosthetic resins’ technical characteristics and their oral biocompatibility.

## 5. Conclusions

The present scientific research analyzes the biochemical interaction between saliva and several interim prosthetic materials (pressure/heat-cured acrylic resin, milled PMMA, 3D printed resin, and pressure/heat-cured micro-filled indirect composite resin). Based on the results obtained and limited to our study, the following conclusions can be drawn:

1. The tested materials do not significantly alter the antioxidant capacity of the incubated saliva;
2. The tested materials do not influence the salivary inflammatory status;
3. Discreet differences between the distribution of the values of the investigated parameters depending on the material type were noticed without being, however, clinically relevant.

## Figures and Tables

**Table 1:** Average value of the biochemical parameters for each material type.

Material/ Parameter (Average ± SD)	Pressure/Heat-Cured Acrylic Resin		Milled PMMA		3D-Printed Resin		Composite Resin	
	Before Incubation	After Incubation	Before Incubation	After Incubation	Before Incubation	After Incubation	Before Incubation	After Incubation
Uric acid/albumin	1.406 ± 0.2	1.11 ± 0.175	2.1 ± 0.2	2.26 ± 0.251	1.6 ± 0.36	1.5 ± 0.2	1.66 ± 0.55	1.66 ± 0.461
GGT/albumin	4.76 ± 1.06	4.866 ± 1.3	4.16 ± 0.305	4.1 ± 0.458	4.66 ± 0.75	4.5 ± 0.984	4.1 ± 0.5	4.066 ± 0.763
IL-6/albumin	91.88 ± 81.95	91.36 ± 81.16	98.47 ± 9.05	95.41 ± 10.53	20.33 ± 9.76	19.3 ± 8.35	46.69 ± 9.302	45.64 ± 8.07
OXSR1/albumin	0.583 ± 0.1	0.476 ± 0.151	0.433 ± 0.081	0.41 ± 0.098	0.593 ± 0.258	0.556 ± 0.228	0.43 ± 0.207	0.493 ± 0.283
TNFα/albumin	0.636 ± 0.145	0.616 ± 0.09	0.383 ± 0.06	0.37 ± 0.06	0.253 ± 0.075	0.25 ± 0.05	0.273 ± 0.055	0.286 ± 0.055
TAC/albumin	1.48 ± 0.137	1.173 ± 0.243	1.63 ± 0.152	1.56 ± 0.23	1.933 ± 0.568	1.833 ± 0.351	2.23 ± 0.75	2.166 ± 0.642

**Table 2:** Comparison of biochemical parameters between material types.

Before Incubation		After Incubation	
Parameter	*p*	Parameter	*p*
Uric acid/albumin	0.192 *	Uric acid/albumin	0.032 **
GGT/albumin	0.683 **	GGT/albumin	0.696 *
IL-6/albumin	0.003 ***	IL-6/albumin	0.004 ***
OXSR1/albumin	0.592 **	OXSR1/albumin	0.850 *
TNFα/albumin	0.003 *	TNFα/albumin	0.001 *
TAC/albumin	0.267 **	TAC/albumin	0.061 **

* One-Way ANOVA Test, ** Kruskal-Wallis H Test, *** Welch ANOVA.

## Data Availability

The data are contained within the article.
